# Appearance of Thiacloprid in the Guttation Liquid of Coated Maize Seeds

**DOI:** 10.3390/ijerph17093290

**Published:** 2020-05-08

**Authors:** Mária Mörtl, Eszter Takács, Szandra Klátyik, András Székács

**Affiliations:** Agro-Environmental Research Institute, National Agricultural Research and Innovation Centre, H−1022 Budapest, Hungary; takacs.eszter@akk.naik.hu (E.T.); klatyik.szandra@akk.naik.hu (S.K.); szekacs.andras@akk.naik.hu (A.S.)

**Keywords:** clothianidin, cross-contamination, guttation liquid, neonicotinoid, seed coating, thiacloprid, thiamethoxam

## Abstract

Thiacloprid (TCL) uptake by maize plants that emerge from coated seeds has been investigated and characterized via measurements of the compound in the guttation liquid. TCL levels were determined in the guttation liquid: (a) under field and semi-field conditions, (b) for different maize varieties, (c) applying different dosages, and (d) as affected by cross-contamination between maize seeds via soil. Cross-contamination was described by uptake interactions between seeds coated with TCL and neighboring seeds not coated or coated with other neonicotinoids, e.g., either thiamethoxam (TMX) or clothianidin (CLO). TCL levels remained under 100 µg/mL in the guttation liquid under field conditions, and were quantifiable even on the 39th day after planting of coated seeds. Higher levels up to 188.6 µg/mL were detected in plants grown under semi-field conditions in pots. Levels in the guttation liquid were also found to be influenced by the applied dosages. The uptake of TCL was found to vary for different maize varieties. Appearance of TCL as a cross-contaminant in the guttation liquid of neighboring plants emerging from non-coated maize seeds indicates translocation of the compound via soil. Peak levels of TCL cross-contamination were found to be lower (43.6 µg/mL) than the corresponding levels in the parent maize plants emerging from coated seeds (107.5 µg/mL), but values converge to each other. Similar trends were observed with neighboring seeds coated with other neonicotinoids (TMX or CLO). The translocation rate of TCL and its uptake by other plants seem to be lower than that of TMX or CLO.

## 1. Introduction

Neonicotinoids are currently among the most widely used insecticides in the world. Their use remarkably increased in the last decades, whereas organophosphates and carbamates have lost market share [1] due to pest resistance developed by different insect species. Nevertheless, selection pressure which originated from the large-scale deployment of neonicotinoids mainly as seed coating inevitably led to development of resistance to these active ingredients (AIs) as well [2,3]. Metabolomic resistance, e.g., enhanced monooxygenase activity [3], appears much more common than mutation of the target site (insect nicotinic acetylcholine receptors) [2]. Due to their extended use and high leaching potential from soils of low organic content [4,5,6], neonicotinoids became ubiquitous contaminants worldwide in surface waters [7]. Low levels, usually only in the low ng/L range, have been detected [8,9,10,11,12], but even these concentrations could affect aquatic species [7,13]. Data confirm that the current overwhelming use of systemic pesticides is not sustainable globally [14]. Application of alternative pest management strategies was suggested recently [15] to eliminate neonicotinoid-based chemical pest control in cropping systems. These tools provide considerable reduction in the use of these insecticides avoiding their prophylactic application as seed coatings. As suitable alternatives, agroecological methods (e.g., mating disruption, habitat manipulation, etc.) after risk assessment and financial compensation systems for unpredicted damages by crop insurance are preferred.

Neonicotinoids in seed treated crops are assumed to contribute to pollinator decline [16] and have adverse effects on other non-target species as well [17]; therefore, the European Commission (EC) restricted the use of clothianidin (CLO), thiamethoxam (TMX) and imidacloprid (IMI) as seed treatment on flowering crops in 2013 [18]. Since these restrictions, the use of another neonicotinoid AI, thiacloprid (TCL) emerged for seed coating of maize. The initial registration of TCL in 1999 was extended to seed coating in 2012, prior to the above restrictions. Formulated plant protection products (PPPs) containing TCL are sold under the brand names of Calypso 480 SC (spray, Bayer) and Biscaya (spray, Bayer), or Sonido 400 FS (coating agent, Bayer). TCL belongs to the N-cyanoamidine group of neonicotinoid insecticides that are much less toxic to bees than N-nitroguanidine compounds [19] due to differences in metabolism by cytochrome P450s of the CYP9Q subfamily. Nevertheless, negative effects of TCL, e.g., to bumblebee colony development under field conditions, have also been reported [20].

Based on a scientific report of the European Food Safety Authority (EFSA) [21], the EC adopted stricter regulations in 2018, and completely banned outdoor use of the three neonicotinoids earlier restricted [22,23,24]. Currently, their uses as spray insecticides or for seed treatment are allowed only in permanent greenhouses, and the crops affected must stay there during their entire life cycle. Furthermore, even in these cases particular attention must be paid to risk mitigation including ground water pollution and exposure of pollinators or aquatic organisms. France went even further and banned these three ingredients along with TCL and acetamiprid (ACE), not only outdoors but in greenhouses too [25]. This year the EC decided not to renew the approval of TCL [26] following the scientific advice by EFSA [27]. The Authority identified a critical concern in relation to the contamination of groundwater by certain metabolites of TCL that may occur above the maximum residue level in drinking water of 0.1 μg/L. It cannot be excluded that these metabolites share the carcinogenic properties of the parent AI TCL, classified as a “likely” human carcinogen based on thyroid and uterine tumors in rats and ovary tumors in mice [28] or a category 2 carcinogen [27]; and also as a compound of category 1B reproductive toxicity [27]. Therefore, even low exposure levels of humans to TCL cannot be considered negligible. PPPs containing TCL can be sold and applied in the European Union until August 2020 and February 2021, respectively.

Neonicotinoids are also under revision in the US by the Environmental Protection Agency (US EPA), initially planned to be completed by 2019 [29]. A detailed review of four neonicotinoids (IMI, CLO, TMX and ACE) is scheduled for 2020 and the proposed interim decisions have been published [30]. Among other restrictions, application of these insecticides to blooming crops and ban of the household use of IMI on residential lawns and turf are proposed. As for TCL, registration has been voluntarily canceled by the registrant [29].

Girolami et al. [31] drew attention to the importance of guttation that might be a novel means of intoxication of bees with xenobiotics. Nevertheless, it is still under discussion whether the guttation liquid indeed represents a relevant exposure route of bees to neonicotinoids and whether this effect alone could exert negative effects on them [32,33]. However, bee colony losses are probably of multi-factorial origin and many other stressors (nutritional shortage, parasitism, residues) possibly contribute. Translocation of neonicotinoid insecticides from coated maize seeds to the guttation drops resulted in high concentrations reaching the level of 100 µg/mL for TMX and CLO [31], and similar data were reported later with levels up to 102 µg/mL for CLO and 146 µg/mL for TMX [34]. Even higher maximum levels (above 100 µg/mL) were determined in an indoor study for TMX and CLO, and in a field study for TMX and IMI in the guttation liquid of maize which emerged from coated seeds [35,36]. Levels of these ingredients in the guttation liquid are indicators for neonicotinoid uptake, and cross-contamination from coated to non-coated maize seeds was also proven to occur [35]. Levels depend on many factors including physico-chemical characteristics of the AI, soil type, meteorological conditions, plant species, developmental stage, etc. Neonicotinoid residues in guttation liquids of other crops were also detected. Significantly lower levels (up to 0.130 µg/mL AI) were released from TMX into the guttation fluid of seed-coated winter oilseed rape that declined to concentrations < 0.030 µg/mL by winter dormancy [37]. IMI was detected at up to 4.1 μg/mL after a soil application and at 37 μg/mL when individual cantaloupe plants were treated with drenches of IMI [38]. An average level of 88 ng/g of IMI residues was determined in turf grass guttation 1 week after treatment, which declined to 23 ng/g within 3 weeks [39]. Systemic ingredients from maize seed coating might be taken up by weeds in close proximity via soil and also appeared in the guttation drops of the creeping thistle or red poppy [40].

The aim of this work was to study the uptake of TCL by maize plants that emerged from coated seeds. To our knowledge, this is the first study to document the levels of TCL in the guttation liquid of maize. We have chosen it as a model species for our investigations as maize shows a high guttation frequency at young growth stages, and large guttation drops are produced even under low relative air humidity. Ingredient uptake was characterized by the levels measured in the guttation liquid of maize: (a) under field and semi-field conditions, (b) for different maize varieties, (c) applying different dosages, and (d) as affected by cross-contamination between maize seeds via soil.

## 2. Materials and Methods

CLO, TCL and TMX analytical standards were purchased from Sigma (Pestanal grade). Methanol was of high performance liquid chromatography (HPLC) grade (Fisher Chemical, Loughborough, UK) and water was purified on an YL Aqua MAX-ultra (YL Instruments, Anyang, Korea) device.

Maize seedlings were obtained from commercially available seeds LG 30.369 (Limanova) that were coated with Sonido coating material containing TCL pesticide AI. We have used 9 different types for comparison of maize varieties under semi-field conditions: 3 commercial (DK 440, DKC 4964 − Die Sandra, GKT 372), 3 Hungarian landrace (Blue, White Kiskun, Mindszenti) and 3 ancient “heirloom” (Dakota black, Strawberry red, Glass gem).

Blue landrace maize seeds were used as blank or non-coated control in cross-contamination experiments, whereas commercially available seeds (Dekalb 449 and Occitan 380) treated with CLO and TMX were used as differently coated seeds.

Seed treatment with neonicotinoids was accomplished in the experiments in three ways: (a) coating by the manufacturer in the case of pre-coated commercial seeds; (b) on-farm coating of uncoated seeds; and (c) uncoated seeds placed in gelatin capsules previously loaded with the corresponding dosages of TCL (in this case only half capsule shells were used to leave the upper end of the capsule open for the seed to germinate and grow in the soil). The AI content of coated seeds was determined according to our previous procedure [35]. Briefly, insecticide content from individual seeds was extracted with 10 mL of methanol using ultrasound agitation for 10 min in 15 replications. Solutions were analyzed after ten-fold dilution with water and filtration through a 0.45 μm hydrophilic polytetrafluoroethylene (PTFE) syringe filter (Labex Ltd., Budapest, Hungary). For CLO (Poncho PRO; Bayer Crop Science) and TMX (Cruiser, Syngenta International) the recommended application rates are 1.25 mg per seed and 0.63 or 1.26 mg per seed, while the measured doses were 1.22 ± 0.66 mg CLO per seed and 0.61 ± 0.07 mg of TMX per seed for Dekalb 449 and Occitan 380 seeds, respectively [35,41].

A clay type soil which originated from Budapest (Hungary), characterized by 12.7% sand, 55.5% silt and 31.8% clay content and 2% of organic matter, was used in all cultivation experiments. Soil was applied to the cultivation pots without any pre-treatment with other agrochemicals (e.g., fertilizers, soil pesticides or disinfectants).

### 2.1. Maize Plantations and Sample Collection

Neonicotinoid uptake by maize plants has been investigated under outdoor conditions in the field or in pots (semi-field). Outdoor cultivation experiments in the field (6 rows, spaced 25 cm within the row and 50 cm between rows) was performed with LG 30.369 in May and June 2015. Different maize varieties were cultivated under semi-field conditions sown in April 2019 into large pots (66 × 44 cm, 90 L). The experimental units consisted of 50 maize seeds per maize variety (5 rows, spaced 6 cm within the row and 7 cm between rows in each pot). Each seed was individually put into a gelatin capsule (open from above) containing the recommended dose of 1 mg/kernel of TCL and the capsules were set. As we have no commercial coating facility to assure the accuracy of the application of seed treatment products for such a low number of seeds, we ensured in this way that each seed received the same treatment. It has to be noted, that this seed treatment is not equivalent to commercial seed coating as the insecticide is not placed directly on the surface of the seed, but allows accurate insecticide dosages in its immediate surroundings. Analysis of the guttation liquids was carried out in combined samples. Sampling regimes were of longer duration, but data are shown for 43 days after sowing. Thereafter, weather conditions prevented sampling, or reliability of data was lost due to precipitation or lower guttation intensity. TCL was detected as an ingredient in the guttation liquid for up to 64 days after planting. If irrigation was needed, it was applied in the afternoon.

The other two experiments started in the middle of August 2015 to study either the dose dependence of the levels in the guttation liquid or the cross-contamination between plants from coated and non-coated seeds. They were carried out outdoors in small pots (12 cm diameter, 1 L volume, without drainage holes) containing 1 or 2 seeds per pot. Maize seedlings were irrigated daily with 50 mL of water per pot. The experimental units consisted of 12 maize plants (or 2 × 12 plants for cross-contamination among seeds). Analysis of the guttation liquids was carried out individually only at the beginning of the sampling regime to identify plants by coating AI. Quantitation has not been found reliable individually due to the low sample volumes, therefore, only the results for combined samples are given.

The effects of dosages were investigated with TCL-coated seeds, containing low (0.054 ± 0.005 mg/kernel TCL) or high (1.16 ± 0.18 mg/kernel TCL) doses of the coating insecticide. Cross-contamination was studied with non-coated seeds or with differently (TMX or CLO) coated seeds. Maize plants which emerged from these seeds were subjected to cross-contamination by neonicotinoid from TCL-coated maize seeds planted in close proximity. A single maize seed per cultivation pot was applied together with a single maize seed coated with TCL. Distances were approximately 5 cm between the two plants.

Upon the emergence of the maize plants, guttation liquid samples were taken from the leaf edges daily using digital pipettes equipped with tips of 100 µL. Outdoor samples were collected once a day between 7 and 8 a.m. because the guttation drops disappeared later (due to evaporation, re-absorption or other causes). Combined samples of each type were subjected to sample preparation consisting of dilution and filtration. If the daily obtained amounts of the guttation liquid did not exceed the volume of 300 µL, the combined samples were diluted as necessary with known amounts of water prior to filtration on a PTFE syringe filter with a pore size of 0.45 µm (Labex Ltd., Hungary). Not more than ten-fold dilution was applied, and actual dilution factors (w/w) were determined. Number of drops per sample varied greatly as the volumes of collected liquid were different. In general, analysis was possible if at least 10 drops could be collected, and 30–50 collected drops allowed analysis without dilution prior to filtration. Samples collected were stored at 4 °C until HPLC analysis. Sampling periods varied depending on guttation intensity and meteorological conditions.

### 2.2. HPLC Analysis

Analyses of samples were performed on a YL9100 HPLC system equipped with a YL9150 autosampler (YL Instruments, Korea) and a C18 column (150 mm × 4.6 mm i.d., 5 µm) at 40 °C. The method developed earlier [4] was used for determination of TMX and CLO. This method was modified in order to detect other ingredients from the coating material of LG 30.369 seeds as follows. UV detector signals were recorded at λ = 252 and 269 nm for TCL. Peak purities were systematically checked by recording absorption at two wavelengths, and peak area ratios at those wavelengths were compared to the ratios characteristic of standard solutions of the analyte. Guttation liquids of blank or non-coated control plants did not contain interfering matrix components, therefore quantitation was based on instrumental (external) calibration with standard solutions in the range between 0.050 and 150 µg/mL. Recoveries of the sample preparation and HPLC analysis process were evaluated using guttation liquid of blank samples (maize grown from uncoated seeds with no exposure to neonicotinoids) spiked with TCL at known concentrations. Thus, TCL was not detected in the blank (not spiked) sample, and recovery values with spiked samples at TCL concentrations between 0.05 and 2.00 µg/mL ranged between 72% and 108% without apparent dose dependence. The linear regression value of calibration curves was 0.9976 and the slope was 82.93 for TCL. The ratio of signal intensities (peak areas) recorded at 252 nm and at 269 nm was found to be 2.32 for standard solutions of TCL. Standard deviation calculated from the three parallel injections of standard solutions ranged between 0.3 and 1.7%. The eluent flow rate was 1.0 mL/min with initial eluent composition (65:35 = A:B eluents, A = 90% water: 10% MeOH, B = MeOH), held for 1 min, followed by a linear gradient (to reach 85% MeOH at 3.5 min, held till 8 min). The limit of detection (LOD), determined with standard solutions and in guttation liquid matrices was 0.030 µg/mL for TCL in both cases. Analyte concentrations in the guttation liquid samples were calculated with the corresponding applied dilution factor (see sample preparation, above) considered. The retention time was 5.12 min for TCL.

## 3. Results

### 3.1. Apperance of TCL in Guttation Liquid of Maize Cultivated under Field Conditions

**Figure 1:**
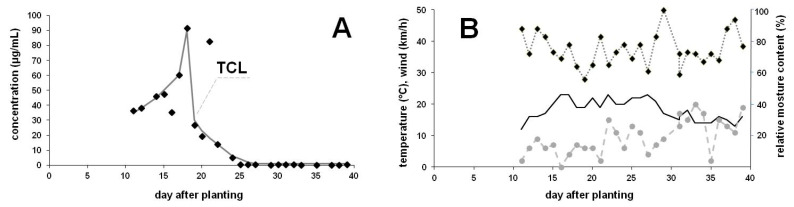
Neonicotinoid thiacloprid (TCL) occurrence from the seed coating in a commercial maize variety (LG 30.369) cultivated under field conditions (sown in May). (**A**): Concentration of TCL (µg/mL) in the guttation liquid of maize as a function of time after sowing. (**B**): Meteorological parameters during the sampling period recorded at 7:30 a.m. each day. Relative moisture content (%, ♦ dotted line), temperature (°C, continuous line) and wind speed (km/h, • dashed line).

Concentrations measured in the guttation liquid of TCL-coated maize under field conditions and corresponding meteorological conditions recorded during the sampling period in Budapest are summarized in Figure 1. Guttated TCL levels rose until the 18th day after planting (18th May), then decreased, although certain outlier values were also detected. After the 25th day only low levels were observed, ranging between 69 and 482 ng/mL with an average level of 242 ng/mL, but even in the sample collected on the 39th day, TCL could be quantified at a level of 238 ng/mL. In this last period, variation in concentrations is characterized by slow decline with fluctuations.

### 3.2. Effect of Maize Varieties on the TCL Levels in Guttation Liquid

**Figure 2:**
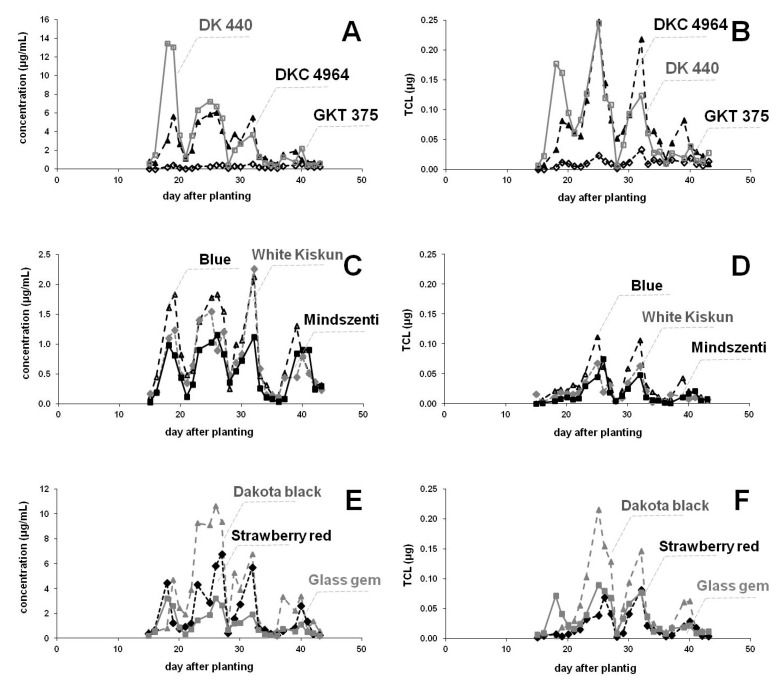
Neonicotinoid (TCL) occurrence from the seed coating in commercial (**A**,**B**; DK 440 **□**, DKC 4964 − Die Sandra ▲, GKT 372 **◊**), Hungarian landrace (**C**,**D**; Blue Δ, White Kiskun **♦**, Mindszenti ■) and „heirloom” (**E**,**F**; Dakota black **▲**, Strawberry red ♦, Glass gem **■**) maize varieties cultivated under semi-field conditions (sown in April). Concentration of TCL (**A**,**C**,**E**; µg/mL) and average amount of TCL (**B**,**D**,**F**; µg) excreted by a single plant in the guttation liquid of maize as a function of time after sowing.

TCL levels in the guttation liquid were compared among maize varieties cultivated under semi-field conditions in the spring. Maize varieties compared under semi-field conditions are listed in Table 1. Concentration of TCL and average amount of TCL excreted by a single plant as a function of time after sowing are shown in Figure 2. Typical concentrations in seed treatments in gelatin capsules were found lower than those observed for coated seeds, presumably due to the less intensive contact between the seed and the AI. There were some parallel fluctuations observed that can be explained by changes in meteorological conditions.

Among the commercial seeds the behavior of DK 440 and DKC 4964 − Die Sandra was similar, but GKT 372 took up the smallest amount of TCL from the investigated maize sorts. Ingredient uptake and levels were less in the guttation drops of Hungarian landrace (Blue, White Kiskun, Mindszenti) varieties compared to commercial seeds DK 440 and DKC 4964 − Die Sandra, but more than that of GKT 372. Within the landrace group levels were similar. There were also differences among the three ancient “heirloom” (Dakota black, Strawberry red, Glass gem) varieties. Data for Dakota black were comparable with commercial seeds DK 440 and DKC 4964 − Die Sandra, whereas concentrations determined for Glass gem were closer to the values measured for the Hungarian landrace group. It is worthy of note that concentration is influenced not only by the ingredient content excreted, but also by the amount of guttation drops produced. For example, concentration in the guttation liquid of Glass gem was the lowest within the group, but the average amount of TCL excreted by a single plant is the lowest for the Strawberry red variety as it exuded almost half as much guttation liquid than Glass gem.

### 3.3. Effect of TCL Doses in Seed Coating on the Levels in Guttation Liquid of Maize

**Figure 3:**
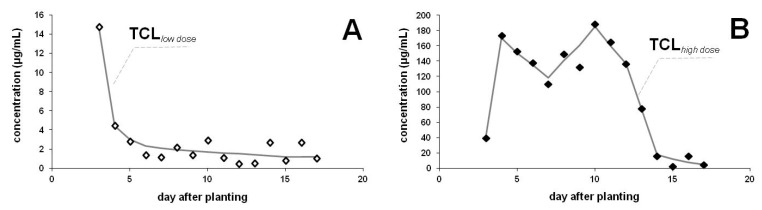
Neonicotinoid (TCL) occurrence from the seed coating in a commercial maize variety (LG 30.369) coated with different neonicotinoid dosages (**A**: **◊** low dose, **B**: ♦ high dose) cultivated under semi-field conditions (sown in August). Concentration of TCL (µg/mL) in the guttation liquid of maize as a function of time after sowing.

Levels in the guttation liquid were compared between commercial maize seeds and other seeds containing low doses of TCL. Ingredient contents of coated seeds have been checked by extraction of TCL from individual seeds. The recommended dose for TCL is 1.0 mg/kernel (Sonido; Bayer Crop Science), while the average value measured for commercial and on-farm coated (low dose) seeds were 1.16 ± 0.18 and 0.054 ± 0.005 mg TCL per kernel, respectively. Comparison of the results obtained from parallel plantations of two seeds coated with different dosages (Figure 3) clearly demonstrate that lower doses result in lower levels of TCL in the guttation fluid. These plants were cultivated in a hot summer (August), therefore evaporation of guttation drops resulted in probably higher levels than the typical values in April or May. Also, the sampling period was shorter (17 days), due to the dynamic development of maize plants and because the intensive evaporation of drops prevented longer sample collection. Peak values for commercially purchased seeds exceeded the value of 180 µg/mL, whereas the highest concentration obtained for seeds coated with low doses was as low as 14.7 µg/mL. For plants which emerged from on-farm coated seeds, concentration in the guttation liquid decreased rapidly and from 6th day on levels are characterized by fluctuations rather than a decline. Low levels were observed in this period ranging between 0.46 and 2.69 μg/mL with an average value of 1.51 μg/mL. For plants which emerged from commercially coated seeds, after the first detection of TCL in the guttation on the 3rd day, its levels were detected in the high level region between 110 and 188 μg/mL until the 13th day, when a rapid decline was observed. In sum, less than 1.5% of the initial seed-applied AI was recovered in the guttation liquid.

### 3.4. Cross-Contamination

**Figure 4:**
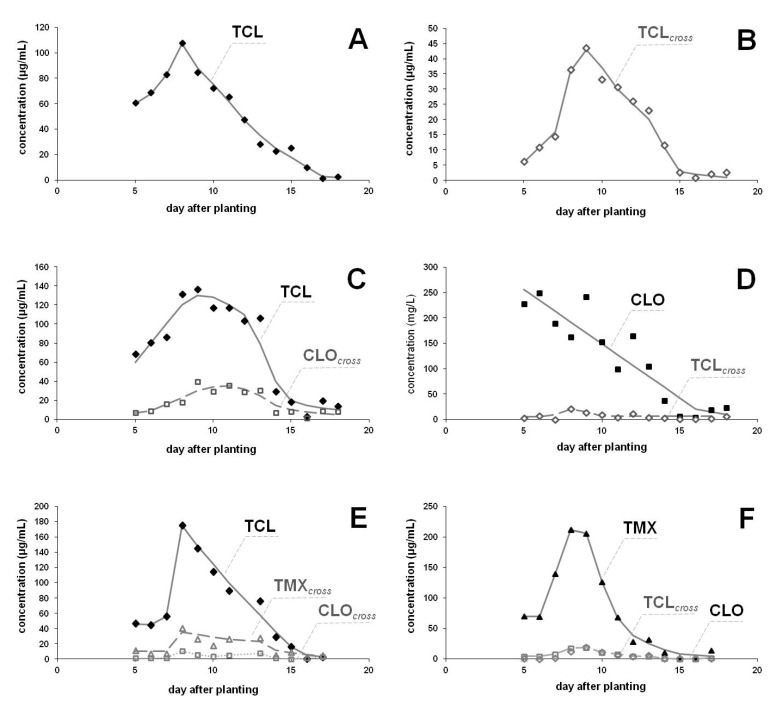
Neonicotinoid occurrence in the guttation liquid from own and neighboring seed coating in commercial (LG 30.369, Occitan, Dekalb) and landrace (Blue) maize varieties cultivated under semi-field conditions (sown in August). Seeds were coated with TCL (**A**,**C**,**E**) at 1.16 ± 0.18 mg/kernel concentration, non-coated (**B**), or coated with CLO (**D**) or TMX (**F**) at 1.22 mg or 0.61 mg per seed concentration, respectively. TCL in plants emerged from seeds coated with TCL (♦) or otherwise coated (**◇**). CLO in plants emerged from seeds coated with CLO (■) or otherwise coated (**□**). TMX in plants emerged from seeds coated with TMX (▲) or otherwise coated (**Δ**). Cross-contamination by coating neonocotinoids from neighboring seeds are indicated in grey color and term “cross” indexed.

Cross-contamination between the plants coated with TCL and with non-coated or those coated with a different neonicotinoid (CLO, TMX) was studied by planting differently treated maize seeds in close proximity to each other. Translocation of neonicotinoids was observed in all cases. Results obtained for ingredient content in the guttation liquids are summarized in Figure 4. The peak levels were 107, 136 and 175 µg/mL, when the seeds were coated with TCL, while maxima reached the level of 200−250 µg/mL for other insecticides. Of course, lower levels were detected for ingredients translocated from neighboring seed coating materials, but their concentrations approached the level of the original coating AI at the end of the sampling period (after two weeks). The peak levels for CLO and TMX in the guttation liquids of plants which emerged from TCL-coated seeds are similar (about 40 µg/mL) in both cases (Figure 4C,E), while the corresponding TCL concentrations (Figure 4D,F) are somewhat lower (around 20 µg/mL). Translocation of TCL to non-coated maize was more effective; the peak level measured in the guttation liquid of maize which emerged from non-coated seeds was as high as 43.6 µg/mL, while the corresponding peak value for TCL-coated seeds reached the level of 107.5 µg/mL. CLO was detected in all samples where TMX was present (Figure 4E,F), as it is a metabolite of TMX with its own insecticidal effect. As we have previously also observed, CLO content may reach 20−30% of the level of TMX due to metabolic transformation in plants, and its levels were similar to those of TCL translocated from the coated seed nearby (Figure 4F).

## 4. Discussion

### 4.1. TCL Levels in the Guttation Liquid of Maize Cultivated under Field Conditions

Concentrations determined for TCL in the guttation liquid of outdoor cultivated maize seedlings (Figure 1) are similar to that observed earlier for plants which emerged from TMX- or CLO-coated seeds [35,40] and the typical values were closer to those that we have detected earlier for CLO-coated maize plants. Extreme values measured at the 16th, 18th and 21st days often occur in outdoor experiments, as guttation is a complex phenomenon affected by many internal and external factors [42]. Exact neonicotinoid levels also depend on meteorological conditions. Weather conditions in the sampling period at 7:30 a.m. in the morning are indicated in Figure 1B. Among the meteorological parameters, high atmospheric humidity is an essential prerequisite, and the temperature and/or wind speed also have dominant roles in the regulation of guttation. Cool nights following warm days provide ideal conditions for the appearance of droplets, but very low temperature stops the root absorption of water. Our experience also confirms that the higher the relative moisture, the higher is the amount of the guttation liquid. Larger amounts of fluid obtained in saturated air resulted in lower concentrations. Due to evaporation of the drops, higher levels were observed when the air moisture level was low, and mild wind also promoted this process. Therefore, on these days (low air humidity and slight air motion) extremely high neonicotinoid levels are expected in the guttation. Higher wind speed together with low relative moisture could prevent collection of the guttation liquid as the droplets may fall down or completely disappear due to the intensive evaporation. Wind speed was typically low (below 10 km/h) in the first period, but later reached 15−20 km/h. Relative moisture content in the mornings varied between 56 and 100%. In Figure 1A two higher outlier values can be observed (on the 18th and 21st days); on both days only low amounts of drops were available. On the 18th day (5th June) low moisture content (64%) and light breeze (7 km/h) certainly elevated the concentration of TCL. On the 21st day weather parameters were not so extreme (2 km/h and 83%), but locally a gentle breeze was experienced. A somewhat lower value was measured on the 16th day, when the moisture content was 69% and no wind was recorded. Of course, other trendlines can also be drawn, as decrease in concentrations are often characterized by fluctuations in outdoor experiments. TCL was easily detectable on the 39th day after planting indicating the long-term presence of the AI in the plant tissues.

### 4.2. Effect of Maize Varieties on the TCL Levels in the Guttation Liquid

Results obtained for different maize varieties (Figure 2) showed considerable differences in the ingredient uptake and in the appearance of the AI in the guttation liquid. Nevertheless, strict borders between the three categories (commercial, landrace, “heirloom”) cannot be marked. Concentrations of TCL for some varieties were higher, but only the landrace group showed uniform behavior among the groups studied. Regarding the daily average amount of TCL excreted by a single plant, values of the landrace group were comparable with those determined for Glass gem and Strawberry red, whereas values for Dakota black were closer to commercial varieties DK 440 and DKC 4964−Die Sandra. Surprisingly, low amounts of TCL appeared in the guttation drops of GKT 372, despite the fact that it produced the highest average volume (44.8 µL/plant/day) of guttation excreted by a single plant during the sampling period. The typical average volumes were around 30 μL, but Dakota and Strawberry red produced only 18.1 and 16.2 μL per plant (see Table 1). Smaller seeds produced less guttation liquid for 5 of 9 maize types studied, but, e.g., Glass gem produced one and a half times more liquid than White Kiskun of the same size, whereas White Kiskun excreted as much as Mindszenti, despite that the seeds of the latter variety are over twice as large in mass. The observed fluctuations are related to varying meteorological conditions. As discussed above (Section 4.1.), exact levels of AIs in the guttation liquid depend on the meteorological parameters. Among them the role of relative humidity and wind speed seemed to be the most important factors. The effect of temperature appears to be less significant, but cool nights after enduringly warm days occur to trigger the formation of guttation liquid on the following dawn. Moreover, soil moisture is also an important prerequisite. Outlier values are expected when suddenly increased relative humidity prevents intensive evaporation of the drops exuded resulting in dilute solutions, whereas dry conditions together with a mild air motion promote intensive evaporation resulting in more concentrated guttation liquids. Unfortunately, weather was unfavorable in the spring of 2019 which influenced the development of the plants and the amount of the guttation liquid produced.

Reported values for the estimated dissipation half-lives (DT_50_s) for TCL in soil vary in a broad range, but typically fall between 3 and 74 days [43]. The fact that TCL was detected up to 64 days after planting indicates a long-term presence of this AI exuded from the plant tissues into the guttation liquid.

### 4.3. Effect of the Applied Dosages

Despite the fact that the coating material is present in a solid form on the surface of the seeds, low doses result in lower AI levels in the guttation liquid (Figure 3). Higher application rates probably result in higher AI levels in the root and shoot tissues, manifested also in the corresponding AI levels in the excreted drops. According to an earlier report [44], highly variable (greater or lower) proportions of a higher initial seed treatment with CLO translocated to the plant tissues were experienced in different years. Peak levels of TCL in the guttation liquid of plants emerged from commercial seeds (coated with the recommended dosage of 1 mg/kernel) were somewhat higher in August and September than that obtained for outdoor plantations in May and June. The relative moisture content was presumably lower in August than in the spring, and higher temperature also enhanced the rate of evaporation resulting in higher concentrations in the guttation liquids collected. Results are certainly influenced by irrigation as soil moisture plays an important role in AI uptake and leaching of systemic pesticides. All these factors contributed to the concentrations determined for samples collected from maize leaves, therefore results are comparable only in those cases when the plants were cultivated under the same conditions. Nonetheless, our results clearly indicate that for this AI the dose also plays an important role in the levels observed in the guttation drops. In addition, the ingredient uptake seems to be prolonged for higher dosages, but here the low water solubility of TCL (185 mg/L) and its retention by soil probably also contribute to the observed differences. In our opinion an ingredient of high water solubility in a soil with low retention capability (e.g., sandy soil) would remain for a shorter period in this high level zone due to the higher leaching rate. Some unknown factors originating from the seed coating technology might also influence the results. In our experiments, 1.21% and 1.08% of the TCL applied at low and high doses, respectively, were excreted into the guttation liquid during the sampling period. These values are similar to our previous findings [35]. Similar low translocation rates were reported earlier [44], when a maximum of 1.34% and 0.26% of the initial seed treatment was recovered from shoot and root tissues of maize.

### 4.4. Cross-Contamination

The measured levels and the observed trends (Figure 4) are in accordance with our earlier results [35]. We have previously described cross-contamination with neonicotinoids from neighboring maize seeds coated with TMX and/or CLO [35] and between maize seeds coated with TMX or CLO and neighboring weeds (red poppy or creeping thistle) [40]. It is worthy of note that in each experiment, including the cross-contamination assessment, non-coated controls have also been sampled to avoid experimental misinterpretation due to matrix interferences. Cross-contamination from the neighboring seeds occurs via soil. The effect of soil quality on neonicotinoid emergence has also been evidenced with three different soil types (sandy, clay, loam): diffusion of TMX or CLO sprayed on the soil surface and their occurrence in the guttation liquid of maize plants which emerged from non-coated seeds was demonstrated [35].

The overall amount of TCL coating material excreted into the guttation liquids of plants which emerged from TCL-coated maize seeds ranged between 0.70 and 1.48%, whereas 1.51% of CLO- and 1.33% of the TMX-coating material appeared in the guttation liquid and an additional 0.12% was detected as CLO metabolite during sampling. Translocation rates of TCL from neighboring TCL-coated seeds were in the range of 0.05 and 0.26%, and the corresponding value was 0.3% for CLO, and 0.38% for TMX including the CLO metabolite. Thus, there were only slight differences experienced between the AIs regarding the cross-contamination rates that can be explained by different water solubility and spreading rates. If the seeds are set further away, diffusion of the AI in the soil results in lower local levels of the cross-contaminating substance, which results in lower ingredient uptake and cross-contamination rates. Translocation rates and appearance of other AIs in the guttation liquid are also influenced by the retention properties of the soil type. Spreading of pesticide AI depends not only on water solubility but is also affected by organic matter and clay content of the soil [4]. Most rapid spreading is expected in sandy soils with low organic matter, while ingredients including neonicotinoids are more strongly retained by the soils with high organic material content [4,5,6]. It is worthy of note that we could not detect TCL in the guttation liquid of maize plants which emerged from non-coated seeds in loam soil after spraying the neonicotinoid ingredient to a soil surface characterized by high organic content (40%<) prior to emergence. In contrast, TMX and CLO appeared after three weeks in the guttation liquid of plants which emerged from the same soil type (loam), while they occurred already in the first appearance of guttation liquid of plants which emerged in sandy soil [35].

## 5. Conclusions

Levels in the guttation liquid of maize seeds coated with TCL are quantifiable even one month after planting of coated seeds under field conditions. Concentrations of this systemic insecticide AI are similar to those that we obtained for TMX- or CLO-coated maize seeds, but peak values were slightly lower compared to those other two neonicotinoid AIs. There were significant differences in the TCL uptake by various maize varieties. Among the types studied the Hungarian landrace group showed the most unique behavior, whereas on the basis of the data determined other varieties studied cannot be categorized into distinct groups. Levels in the guttation drops are also influenced by the applied dosages, but the effect of the applied coating technology cannot be excluded. Appearance of TCL in the guttation liquid of plants which emerged from non-coated maize seeds indicates its translocation via soil. Although peak levels were found to be lower, than those of corresponding coated seeds, values appear to converge to each other. Similar trends were observed during parallel measurements with otherwise coated seeds (TMX or CLO). Slightly lower peak levels were detected for TCL compared to the other two neonicotinoid insecticide AIs, so the translocation rate of TCL and its uptake by other plants are lower than those of TMX or CLO. The observed low translocation efficacy of this targeted pesticide delivery system (i.e., seed coating) together with the high mobility of these AIs contribute to the wild scale contamination of surface waters by neonicotinoids observed worldwide. Such contamination cases can certainly be prevented by a complete ban of the given AI; however, this could result in the re-introduction or increase in the use of environmentally less favorable substances instead. Alternatively, contamination levels can be reduced by: (a) limiting the use of prophylactic applications of the pesticides (e.g., seed coating) only to necessary agrotechnological cases; (b) minimizing the dosages applied to the actual pest to be controlled; (c) shifting to possibly less harmful insecticides, if available (e.g., anthranilic diamides (ryanoids) or tetronic acid derivatives) and/or preferably to agroecological solutions of pest control.

## Figures and Tables

**Table 1:** Maize seeds used for comparison of varieties.

Variety	Category	Average Mass (g/kernel)	Daily Guttation Intensity (µL/plant/day) ^1^	Daily Excreted TCL (ng/plant/day) ^2^
DK 440	commercial	0.19	36.2	103.5
DKC 4964 – Die Sandra		0.30	30.4	97.3
GKT 372		0.26	44.8	15.3
Blue	landrace	0.29	37.5	42.7
White Kiskun		0.17	27.4	26.4
Mindszenti		0.39	27.5	22.5
Dakota black	heirloom	0.11	18.1	77.2
Strawberry red		0.07	16.2	27.0
Glass gem		0.16	40.4	40.3

**^1^** Calculated average daily volume of guttation liquid (µL) produced by a plant during the 25−27 day sampling period, starting at the occurrence of guttation (typically 11 days) until the 43rd day after emergence. As individual daily guttation volumes were too low to be volumetrically measured (only combined volumes from the number (30−45) of emerged plants were determined) and as daily guttation volumes showed an increasing trend as the plants developed, deviations from the average value could not be defined. **^2^** Calculated from combined TCL levels in the guttation liquid and the daily guttation intensity of the given maize variety.
